# 1-(3,3-Dichloro­all­yloxy)-2-nitro­benzene

**DOI:** 10.1107/S1600536812010070

**Published:** 2012-03-14

**Authors:** Dong-mei Ren, Yong-yi Wang

**Affiliations:** a Security and Environment Engineering College, Capital University of Economics and Business, Beijing 10070, People’s Republic of China

## Abstract

In the title compound, C_9_H_7_Cl_2_NO_3_, the dihedral angle between the benzene ring and the plane of the nitro group is 50.2 (1)°, and that between the benzene ring and the best plane through the dichloro­allyl fragment is 40.1 (1)°.

## Related literature

For the synthesis and applications of the title compound, see: Walker *et al.* (2005 ▶). For bond-length data, see: Allen *et al.* (1987) ▶.
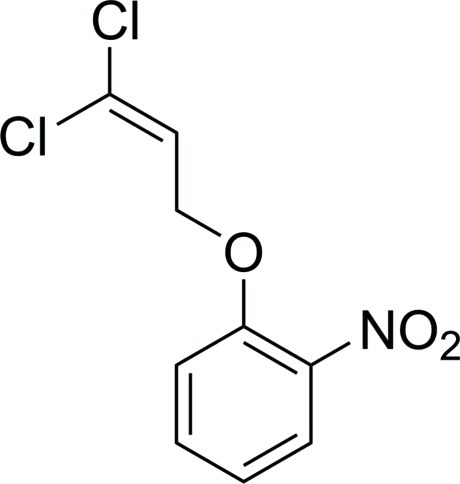


## Experimental

### 

#### Crystal data

C_9_H_7_Cl_2_NO_3_
*M* *_r_* = 248.06
Monoclinic, 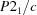

*a* = 4.0210 (8) Å
*b* = 21.506 (4) Å
*c* = 12.333 (3) Å
β = 96.41 (3)°
*V* = 1059.8 (4) Å^3^
*Z* = 4
Mo *K*α radiation
μ = 0.60 mm^−1^
*T* = 293 K
0.30 × 0.20 × 0.10 mm

#### Data collection

Enraf–Nonius CAD-4 diffractometer
Absorption correction: ψ scan (North *et al.*, 1968 ▶) *T*_min_ = 0.841, *T*_max_ = 0.943
4390 measured reflections
1941 independent reflections
1416 reflections with *I* > 2σ(*I*)
*R*_int_ = 0.063
3 standard reflections every 200 reflections intensity decay: 1%

#### Refinement

*R*[*F* ^2^ > 2σ(*F* ^2^)] = 0.050
*wR*(*F* ^2^) = 0.164
*S* = 1.00
1941 reflections
136 parameters
H-atom parameters constrained
Δρ_max_ = 0.36 e Å^−3^
Δρ_min_ = −0.29 e Å^−3^

### 

Data collection: *CAD-4 Software* (Enraf–Nonius, 1985 ▶); cell refinement: *CAD-4 Software*; data reduction: *XCAD4* (Harms & Wocadlo,1995 ▶); program(s) used to solve structure: *SHELXS97* (Sheldrick, 2008 ▶); program(s) used to refine structure: *SHELXS97* (Sheldrick, 2008 ▶); molecular graphics: *SHELXTL* (Sheldrick, 2008 ▶); software used to prepare material for publication: *SHELXTL*.

## Supplementary Material

Crystal structure: contains datablock(s) I, global. DOI: 10.1107/S1600536812010070/vm2161sup1.cif

Structure factors: contains datablock(s) I. DOI: 10.1107/S1600536812010070/vm2161Isup2.hkl

Supplementary material file. DOI: 10.1107/S1600536812010070/vm2161Isup3.cml

Additional supplementary materials:  crystallographic information; 3D view; checkCIF report

## Figures and Tables

**Fig. 1.**
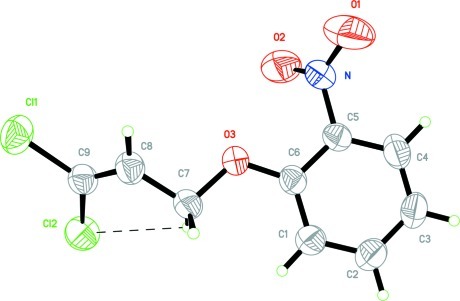
The molecular structure of (I), with the atom-numbering scheme. Displacement ellipsoids are drawn at the 50% probability level.

**Fig. 2.**
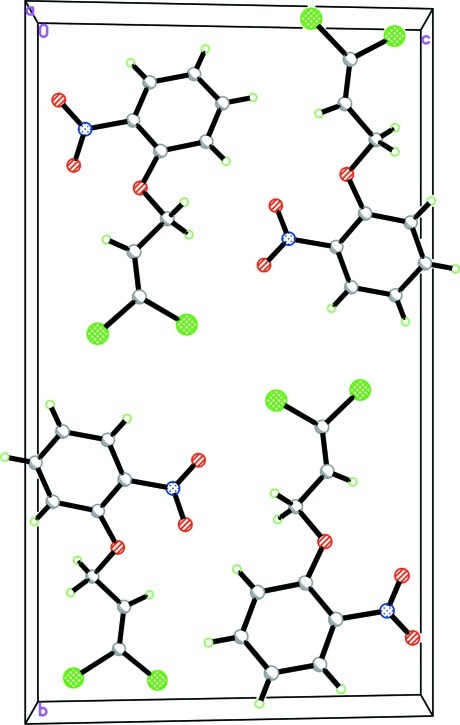
Packing diagram of (I) viewed down the a-axis.

## supplementary crystallographic information

### Comment

The title compound, 1-(3,3-dichloroallyloxy)-2-nitrobenzene is an important intermediate in the synthesis of phenanthrenes (Walker *et al.*, 2005). Here we report here the molecular and crystal structure of the title compound (Fig. 1).

There are no classic hydrogen bonds found, but a short intramolecular contact C7—H7B···Cl2 is observed (C7—H7B: 0.97 Å, H7B···Cl2: 2.700 Å, C7···Cl2: 3.139 (3) Å, C7—H7B···Cl2: 108.00).

The dihedral angle between the benzene ring (C1—C6) and the plane of the nitro group is 50.2 (1)°, and between the benzene ring and the best plane through the dichloroallyl fragment (C7—C9, Cl1, Cl2) 40.1 (1)°.

The packing is shown in Figure 2 and contains a short Cl1···Cl2 (*x* + 1, *y*, *z*) contact (3.6668 (16) Å).

### Experimental

The title compound, (I) was prepared by a method reported in literature (Walker *et al.*, 2005). The crystals were obtained by dissolving (I) (0.1 g) in methanol (30 ml) and evaporating the solvent slowly at room temperature for about 8 d.

### Refinement

All H atoms were positioned geometrically and constrained to ride on their parent atoms, with C—H = 0.93 Å for aromatic H and 0.96 Å for alkyl H, respectively. The *U*_iso_(H) = *xU*_eq_(C), where *x* = 1.2 for aromatic H and *x* = 1.5 for other H.

### Crystal data

**Table:**

C_9_H_7_Cl_2_NO_3_	*F*(000) = 504
*M**_r_* = 248.06	*D*_x_ = 1.555 Mg m^−^^3^
Monoclinic, *P*2_1_/*c*	Mo *K*α radiation, λ = 0.71073 Å
Hall symbol: -P 2ybc	Cell parameters from 25 reflections
*a* = 4.0210 (8) Å	θ = 10–13°
*b* = 21.506 (4) Å	µ = 0.60 mm^−^^1^
*c* = 12.333 (3) Å	*T* = 293 K
β = 96.41 (3)°	Block, colourless
*V* = 1059.8 (4) Å^3^	0.30 × 0.20 × 0.10 mm
*Z* = 4	

### Data collection

**Table:**

Enraf–Nonius CAD-4 diffractometer	1416 reflections with *I* > 2σ(*I*)
Radiation source: fine-focus sealed tube	*R*_int_ = 0.063
Graphite monochromator	θ_max_ = 25.4°, θ_min_ = 1.9°
ω/2θ scans	*h* = 0→4
Absorption correction: ψ scan (North *et al.*, 1968)	*k* = −25→25
*T*_min_ = 0.841, *T*_max_ = 0.943	*l* = −14→14
4390 measured reflections	3 standard reflections every 200 reflections
1941 independent reflections	intensity decay: 1%

### Refinement

**Table:**

Refinement on *F*^2^	Primary atom site location: structure-invariant direct methods
Least-squares matrix: full	Secondary atom site location: difference Fourier map
*R*[*F*^2^ > 2σ(*F*^2^)] = 0.050	Hydrogen site location: inferred from neighbouring sites
*wR*(*F*^2^) = 0.164	H-atom parameters constrained
*S* = 1.00	*w* = 1/[σ^2^(*F*_o_^2^) + (0.1*P*)^2^ + 0.2*P*] where *P* = (*F*_o_^2^ + 2*F*_c_^2^)/3
1941 reflections	(Δ/σ)_max_ < 0.001
136 parameters	Δρ_max_ = 0.36 e Å^−^^3^
0 restraints	Δρ_min_ = −0.29 e Å^−^^3^

### Special details

**Table:**

Geometry. All e.s.d.'s (except the e.s.d. in the dihedral angle between two l.s. planes) are estimated using the full covariance matrix. The cell e.s.d.'s are taken into account individually in the estimation of e.s.d.'s in distances, angles and torsion angles; correlations between e.s.d.'s in cell parameters are only used when they are defined by crystal symmetry. An approximate (isotropic) treatment of cell e.s.d.'s is used for estimating e.s.d.'s involving l.s. planes.
Refinement. Refinement of *F*^2^ against ALL reflections. The weighted *R*-factor *wR* and goodness of fit *S* are based on *F*^2^, conventional *R*-factors *R* are based on *F*, with *F* set to zero for negative *F*^2^. The threshold expression of *F*^2^ > σ(*F*^2^) is used only for calculating *R*-factors(gt) *etc*. and is not relevant to the choice of reflections for refinement. *R*-factors based on *F*^2^ are statistically about twice as large as those based on *F*, and *R*- factors based on ALL data will be even larger.

### Fractional atomic coordinates and isotropic or equivalent isotropic displacement parameters (Å^2^)

**Table:**

	*x*	*y*	*z*	*U*_iso_*/*U*_eq_	
N	−0.0930 (8)	0.17453 (13)	0.1420 (2)	0.0607 (7)	
Cl1	0.6644 (2)	0.46194 (4)	0.18851 (7)	0.0679 (3)	
C1	0.1777 (8)	0.19522 (14)	0.4390 (2)	0.0509 (7)	
H1A	0.2853	0.2241	0.4870	0.061*	
O1	−0.0106 (14)	0.13642 (18)	0.0804 (2)	0.1309 (16)	
Cl2	0.3945 (3)	0.44863 (4)	0.39369 (7)	0.0729 (3)	
C2	0.0679 (9)	0.13945 (15)	0.4779 (3)	0.0591 (8)	
H2A	0.0987	0.1315	0.5525	0.071*	
O2	−0.2241 (10)	0.22293 (15)	0.1110 (2)	0.0977 (10)	
O3	0.2229 (6)	0.26084 (9)	0.28095 (15)	0.0551 (6)	
C3	−0.0868 (9)	0.09520 (15)	0.4083 (3)	0.0612 (9)	
H3A	−0.1589	0.0579	0.4358	0.073*	
C4	−0.1329 (9)	0.10674 (14)	0.2985 (3)	0.0568 (8)	
H4A	−0.2332	0.0771	0.2507	0.068*	
C5	−0.0295 (8)	0.16271 (13)	0.2596 (2)	0.0469 (7)	
C6	0.1265 (7)	0.20796 (12)	0.3279 (2)	0.0430 (6)	
C7	0.4163 (8)	0.30524 (12)	0.3477 (2)	0.0480 (7)	
H7A	0.6064	0.2853	0.3891	0.058*	
H7B	0.2808	0.3250	0.3982	0.058*	
C8	0.5296 (7)	0.35136 (14)	0.2706 (2)	0.0491 (7)	
H8A	0.6086	0.3361	0.2077	0.059*	
C9	0.5280 (8)	0.41198 (13)	0.2834 (2)	0.0495 (7)	

### Atomic displacement parameters (Å^2^)

**Table:**

	*U*^11^	*U*^22^	*U*^33^	*U*^12^	*U*^13^	*U*^23^
N	0.0812 (19)	0.0533 (16)	0.0480 (13)	−0.0130 (14)	0.0079 (14)	−0.0101 (12)
Cl1	0.0832 (6)	0.0472 (5)	0.0752 (6)	−0.0049 (4)	0.0172 (5)	0.0102 (4)
C1	0.0600 (18)	0.0462 (16)	0.0462 (14)	0.0020 (13)	0.0041 (14)	−0.0025 (11)
O1	0.220 (5)	0.112 (3)	0.0666 (17)	0.022 (3)	0.040 (2)	−0.0235 (17)
Cl2	0.1011 (7)	0.0526 (5)	0.0672 (5)	0.0023 (4)	0.0188 (5)	−0.0173 (4)
C2	0.074 (2)	0.0536 (18)	0.0500 (16)	0.0020 (16)	0.0084 (16)	0.0078 (13)
O2	0.135 (3)	0.095 (2)	0.0579 (15)	0.003 (2)	−0.0152 (16)	0.0072 (14)
O3	0.0769 (15)	0.0431 (11)	0.0442 (10)	−0.0160 (10)	0.0011 (10)	0.0008 (8)
C3	0.079 (2)	0.0393 (16)	0.0656 (19)	−0.0027 (15)	0.0118 (17)	0.0086 (13)
C4	0.067 (2)	0.0393 (15)	0.0654 (19)	−0.0041 (14)	0.0134 (16)	−0.0075 (13)
C5	0.0562 (17)	0.0428 (16)	0.0429 (13)	0.0000 (12)	0.0112 (12)	−0.0062 (11)
C6	0.0482 (15)	0.0364 (14)	0.0451 (13)	0.0027 (11)	0.0082 (12)	−0.0041 (10)
C7	0.0560 (17)	0.0402 (15)	0.0464 (14)	−0.0034 (12)	−0.0003 (13)	−0.0040 (11)
C8	0.0521 (17)	0.0419 (15)	0.0537 (15)	−0.0033 (12)	0.0078 (14)	−0.0035 (11)
C9	0.0548 (18)	0.0421 (16)	0.0512 (16)	−0.0006 (12)	0.0042 (14)	−0.0020 (11)

### Geometric parameters (Å, º)

**Table:**

N—O1	1.189 (4)	O3—C7	1.432 (3)
N—O2	1.209 (4)	C3—C4	1.368 (5)
N—C5	1.466 (4)	C3—H3A	0.9300
Cl1—C9	1.723 (3)	C4—C5	1.378 (4)
C1—C2	1.382 (4)	C4—H4A	0.9300
C1—C6	1.390 (4)	C5—C6	1.390 (4)
C1—H1A	0.9300	C7—C8	1.481 (4)
Cl2—C9	1.710 (3)	C7—H7A	0.9700
C2—C3	1.382 (5)	C7—H7B	0.9700
C2—H2A	0.9300	C8—C9	1.313 (4)
O3—C6	1.352 (3)	C8—H8A	0.9300
			
O1—N—O2	122.3 (3)	C4—C5—N	118.1 (3)
O1—N—C5	118.8 (3)	C6—C5—N	119.7 (3)
O2—N—C5	118.9 (3)	O3—C6—C1	124.7 (2)
C2—C1—C6	119.7 (3)	O3—C6—C5	117.4 (2)
C2—C1—H1A	120.1	C1—C6—C5	117.8 (3)
C6—C1—H1A	120.1	O3—C7—C8	105.3 (2)
C1—C2—C3	121.4 (3)	O3—C7—H7A	110.7
C1—C2—H2A	119.3	C8—C7—H7A	110.7
C3—C2—H2A	119.3	O3—C7—H7B	110.7
C6—O3—C7	118.5 (2)	C8—C7—H7B	110.7
C4—C3—C2	119.5 (3)	H7A—C7—H7B	108.8
C4—C3—H3A	120.3	C9—C8—C7	125.6 (3)
C2—C3—H3A	120.3	C9—C8—H8A	117.2
C3—C4—C5	119.4 (3)	C7—C8—H8A	117.2
C3—C4—H4A	120.3	C8—C9—Cl2	124.0 (2)
C5—C4—H4A	120.3	C8—C9—Cl1	122.1 (2)
C4—C5—C6	122.2 (3)	Cl2—C9—Cl1	113.89 (17)
			
C6—C1—C2—C3	1.3 (5)	C2—C1—C6—O3	180.0 (3)
C1—C2—C3—C4	−0.1 (6)	C2—C1—C6—C5	−1.3 (4)
C2—C3—C4—C5	−1.1 (5)	C4—C5—C6—O3	178.9 (3)
C3—C4—C5—C6	1.1 (5)	N—C5—C6—O3	−2.1 (4)
C3—C4—C5—N	−177.9 (3)	C4—C5—C6—C1	0.1 (4)
O1—N—C5—C4	−50.7 (5)	N—C5—C6—C1	179.1 (3)
O2—N—C5—C4	130.1 (4)	C6—O3—C7—C8	170.5 (2)
O1—N—C5—C6	130.2 (4)	O3—C7—C8—C9	136.4 (3)
O2—N—C5—C6	−49.0 (5)	C7—C8—C9—Cl2	0.4 (5)
C7—O3—C6—C1	5.5 (4)	C7—C8—C9—Cl1	−179.8 (2)
C7—O3—C6—C5	−173.3 (3)		

### Hydrogen-bond geometry (Å, º)

**Table:**

*D*—H···*A*	*D*—H	H···*A*	*D*···*A*	*D*—H···*A*
C7—H7*B*···Cl2	0.97	2.70	3.139 (3)	108
